# Acetic and Acrylic Acid Molecular Imprinted Model Silicone Hydrogel Materials for Ciprofloxacin-HCl Delivery

**DOI:** 10.3390/ma5010085

**Published:** 2012-01-02

**Authors:** Alex Hui, Heather Sheardown, Lyndon Jones

**Affiliations:** 1 Centre for Contact Lens Research, School of Optometry, University of Waterloo, 200 University Avenue West, Waterloo, ON N2L3G1, Canada; E-Mail: lwjones@uwaterloo.ca; 2 Department of Chemical Engineering, McMaster University, 1280 Main Street West, Hamilton, ON L8S4L7, Canada; E-Mail: sheardow@univmail.cis.mcmaster.ca

**Keywords:** molecular imprinting, ciprofloxacin, antibiotic, contact lens materials, silicone hydrogel, drug delivery, combination devices

## Abstract

Contact lenses, as an alternative drug delivery vehicle for the eye compared to eye drops, are desirable due to potential advantages in dosing regimen, bioavailability and patient tolerance/compliance. The challenge has been to engineer and develop these materials to sustain drug delivery to the eye for a long period of time. In this study, model silicone hydrogel materials were created using a molecular imprinting strategy to deliver the antibiotic ciprofloxacin. Acetic and acrylic acid were used as the functional monomers, to interact with the ciprofloxacin template to efficiently create recognition cavities within the final polymerized material. Synthesized materials were loaded with 9.06 mM, 0.10 mM and 0.025 mM solutions of ciprofloxacin, and the release of ciprofloxacin into an artificial tear solution was monitored over time. The materials were shown to release for periods varying from 3 to 14 days, dependent on the loading solution, functional monomer concentration and functional monomer:template ratio, with materials with greater monomer:template ratio (8:1 and 16:1 imprinted) tending to release for longer periods of time. Materials with a lower monomer:template ratio (4:1 imprinted) tended to release comparatively greater amounts of ciprofloxacin into solution, but the release was somewhat shorter. The total amount of drug released from the imprinted materials was sufficient to reach levels relevant to inhibit the growth of common ocular isolates of bacteria. This work is one of the first to demonstrate the feasibility of molecular imprinting in model silicone hydrogel-type materials.

## 1. Introduction

As the contact lens industry continues to grow and develop, novel uses and applications of contact lenses are constantly being contemplated and investigated. Contact lens materials as a vehicle for sustained ophthalmic drug delivery to the eye has had a renewal of interest in the past decade, mainly due to the advent of silicone hydrogel materials, which provide sufficient oxygen delivery to the eye to permit hypoxia-free wear during overnight use [1]. Indeed, in the original patents and designs of soft contact lens materials, the concept of using contact lenses as a reservoir for drugs delivered to the eye was noted, although little work investigating this application has been conducted for over thirty years [2]. Recently, there has been an explosion in the number of studies and groups who have demonstrated an interest in the development of contact lens drug delivery materials. The rationales for the use of contact lenses as drug delivery devices are numerous. First, contact lenses are arguably the most successful biomaterial currently available, with estimates of over 140 million wearers worldwide [3], and are thus firmly embraced by patients and, more importantly, practitioners. Second, contact lenses have already been demonstrated to successfully correct refractive errors in patients. The addition of drug delivery to this correction of refractive error can potentially increase the quality of life in patients by decreasing dosing frequency, while also potentially increasing compliance rates in acute or chronic ophthalmic treatment. Third, there is some evidence that concurrent contact lens and topical ophthalmic treatment is more effective than topical treatment alone. Use of contact lenses has been demonstrated to increase the residence time and/or increase ocular penetration of topically administered agents [4,5]. Use of contact lenses may thus decrease the amount of drug needed to successfully treat ocular disease in patients. Finally, there are many situations or locales around the world where access to pharmacological therapy is inconsistent at best, necessitating the use of treatments that can be administered at a single time and have a long lasting effect. Development of these devices to combat these medical challenges is thus warranted and potentially useful.

There are several clinical scenarios in which a contact lens is already used medically to aid the healing of a patient, with topically prescribed agents being used concurrently with contact lenses. For example, following photorefractive keratectomy (PRK), an ocular laser surgical method used for the correction of refractive error, a bandage contact lens is used for several days post-surgery, due to the absence of the corneal epithelium, which is removed during the course of the procedure [6]. Antibiotic drops are used on top of the lens prophylactically to prevent any post-surgical infection. In patients who present with a traumatic corneal abrasion, a bandage contact lens is often used to increase the rate of healing, while also providing symptomatic pain relief. These patients are often prescribed an antibiotic agent, either prophylactically or to treat any current infection sustained during the trauma. It is evident that if the bandage lens was concurrently providing the symptomatic relief as well as the release of the prophylactic antibiotic agent, then the patient could be permitted to rest and recuperate rather than worrying about drug dosing schedules.

The extended release of drugs from soft contact lens materials (hydrogels) is unfortunately not that simple. Previous studies have demonstrated that commercially available lenses soaked in ophthalmic pharmaceuticals are capable of releasing clinically relevant amounts of drugs, but the release times from these materials is in the order of only minutes to hours [7,8,9,10]. Furthermore, these materials are not designed for extended wear, so even if long term release was achieved, the hypoxia of the cornea that would occur with extended wear would necessitate their removal. Thus, strategies to optimize release times to be more on the order of days or even weeks are needed, if these devices are to be used and marketed effectively.

Numerous strategies have been investigated to slow and/or control the release of pharmaceuticals from contact lenses. Some investigators have found the addition of a diffusion barrier could impede the movement of the drug out of the lens, thus slowing the release. In recent studies investigating this concept for the delivery of dexamethasone and timolol [11,12], vitamin E was used as a diffusion barrier and the authors were able to demonstrate sustained release from these materials for days to weeks, with the time for release being controlled by the amount of vitamin E used. This technique may prove particularly beneficial as it can be used with commercially available materials, thus shortening the regulatory approval processes. Other authors have proposed the use of a drug-impregnated coating on the surface of the lens, using cyclodextrins, nanoparticles or liposomes [13,14,15]. This strategy may be particularly useful for drugs with poor solubility in aqueous environments, as the microenvironment of the coating can be different from the rest of the lens.

One of the more successful strategies in generating extended release times from contact lens materials has been molecular imprinting. Molecular imprinting is a polymerization strategy in which a molecule of interest is present within the pre-polymerization solution of a polymer. The addition of other molecules known as functional monomers, which serve to interact with the functional groups of the template molecule, create “cavities” or “molecular memory” within the material after polymerization is complete [16]. These “cavities” specifically interact with the template molecules, slowing the diffusion of the templates out of the material into solution, and thus extending release times [17]. This technique was originally designed for highly crosslinked, hard plastics for the specific removal of components out of solutions [16]. The challenge has been to adapt this technique for contact lenses, in which a highly crosslinked, rigid type material would not be useful. Despite these challenges, several recent papers have shown this technique to be applicable to the creation of contact lens materials to deliver anti-glaucoma, antibiotic, antihistamine, non-steroidal anti-inflammatory agents (NSAIDs) and wetting agents [17,18,19,20,21]. The gains in delivery time for materials created using this concept have been substantial; whereas non-modified materials may release for only a few hours at most, delivery from imprinted materials in the order of several days have been achieved [21]. Several key insights have been gleaned from previous authors. First, the choice of the template and functional monomer is crucial. There has to be an appropriate interaction between the template and functional monomer to efficiently create the cavities to be fixed during the polymerization process [21]. Second, the amount of functional monomer relative to the template in the polymerization mix is also important. A low functional monomer:template will yield an insufficient number of cavities being created around the template; a too high functional monomer:template ratio will lead to inefficient creation of cavities, as much of the functional monomer will not have the opportunity to interact with the template [19]. Much of the work to-date on imprinted molecules have involved “conventional” higher water content hydrogel materials based on poly-hydroxyethyl methacrylate (pHEMA) [20,22], but more recent work has been performed on the more oxygen permeable siloxane-based hydrogels [23].

Ciprofloxacin-HCl is a second generation fluoroquinolone antibiotic. It interferes with bacterial DNA gyrase, preventing bacterial DNA replication [24]. It is a broad spectrum antibiotic, with activity against both gram-negative and gram positive bacteria [25,26]. It is used ophthalmically as either an eye drop or as an ointment. It is commonly used as a treatment for bacterial conjunctivitis, and is one of only a few drugs that have United States Food and Drug Administration (FDA) indications for the treatment of bacterial ulcers/microbial keratitis [27,28]. Ciprofloxacin exhibits poor aqueous solubility at physiological pH due to its overall neutral charge as a zwitterion at this pH, and the presence of its dual aromatic rings [29]. Its solubility in aqueous media is greatly enhanced in acidic or basic solutions, leading to commercially available ophthalmic preparations having a pH of approximately 4.0, which may cause some stinging or irritation upon instillation [28,29]. When dissolved in high concentrations, ciprofloxacin solutions have a yellowish colour. During a severe infection, the dosing of ciprofloxacin can be as frequent as two drops every fifteen min. This high dose and long term use, coupled with poor solubility of the drug at physiological pH, can lead to the development of white, crystalline precipitates in the cornea or inferior conjunctival sac, although this does not necessarily indicate the need to discontinue treatment [30].

In this current study, molecular imprinting techniques were used to create model silicone hydrogel materials for the delivery of the antibiotic ciprofloxacin-HCl. Acetic and acrylic acid were used as functional monomers, and the effect of functional monomer:template ratio, overall functional monomer concentration and drug loading concentration were all investigated and explored. This study is one of the few studies investigating the use of silicone hydrogel-type materials for the delivery of pharmaceuticals using a molecular imprinting strategy.

## 2. Results and Discussion

### 2.1. Pilot Study: Ciprofloxacin pHEMA-Methacryloxypropyltris (Trimethylsiloxy) Silane (TRIS) Materials with Acetic Acid Functional Monomers

The water content and dry weight of the different acetic acid imprinted model materials is detailed in Table 1. Model lenses created would all be classified as being of low water content, and would require some increase in water content if they were to be used as actual contact lenses on the eye. There was no statistically significant difference between the pHEMA-TRIS-Acetic Acid controls and the pHEMA-TRIS-Acetic Acid Ciprofloxacin imprinted materials, based on a one way analysis of variance (ANOVA) (*p* > 0.05).

**Table 1:** Dry weight and water content of Acetic Acid Imprinted pHEMA-TRIS Materials.

Model lens type	Dry weight (g) (Average ± SD)	Water content (%) (Average ± SD)	Centre thickness (mm) (Average ± SD)	Volume (mm^3^) (Average ± SD)
pHEMA + TRIS + 1% by weight Acetic Acid Control	0.0457 ± 0.0089	15.5 ± 2.7	0.87 ± 0.12	68.1 ± 9.3
pHEMA + TRIS + 1% by weight 4:1 Acetic Acid:Ciprofloxacin	0.0428 ± 0.0078	14.8 ± 2.5	0.93 ± 0.16	73.2 ± 12.9
pHEMA + TRIS + 1% by weight 8:1 Acetic Acid:Ciprofloxacin	0.0396 ± 0.0059	16.7 ± 2.1	0.99 ± 0.14	77.3 ± 10.8

**Figure 1:**
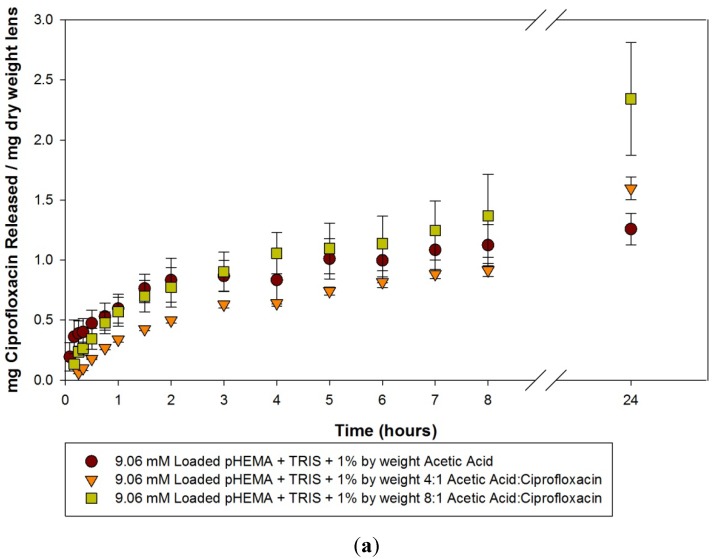

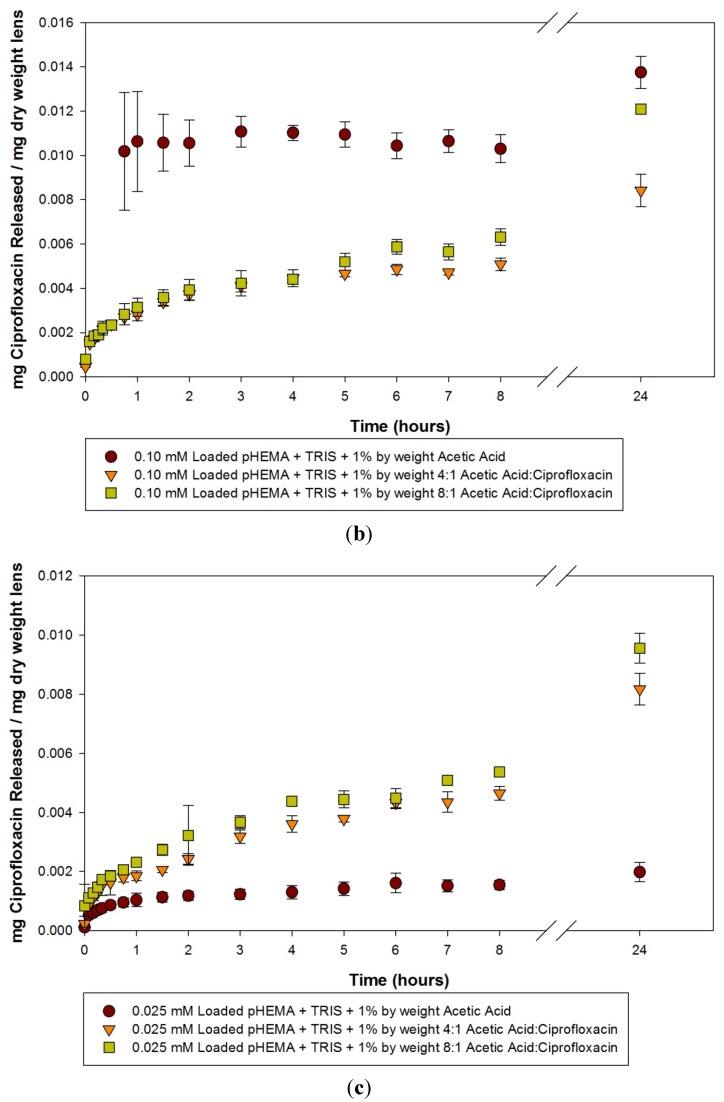
(**a**–**c**) Release curves from acetic acid imprinted materials loaded with (**a**) 9.06 mM ciprofloxacin; (**b**) 0.10 mM ciprofloxacin and (**c**) 0.025 mM ciprofloxacin over 24 h. Values plotted are means ± SD.

The release curves from these materials loaded with 9.06 mM, 0.10 mM and 0.025 mM ciprofloxacin over the first 24 h are seen in Figure 1(a–c). There was no statistically significant difference seen between the imprinted and control model lenses loaded with 9.06 mM, over the course of the 24 h (*p* > 0.05). The initial release from the 0.10 mM and 0.025 mM model lenses are of interest. For 0.10 mM loaded model lenses, the control exhibited a very fast release and almost immediate plateau, at a level higher than the two imprinted materials. For the 0.025 mM loaded model lens, the control model lens again almost immediately reached its final plateau level, but in this situation it was at a level that was below that of the two imprinted materials. Whether this was caused by some residual loading solution on the 0.10 mM loaded discs is unknown. For the imprinted materials, for both the 0.10 mM and 0.025 mM loaded model lenses, there was a slow release of ciprofloxacin into solution over the course of the 24 hours, but there was no statistical significance between the 4:1 and 8:1 imprinted materials.

**Figure 2:**
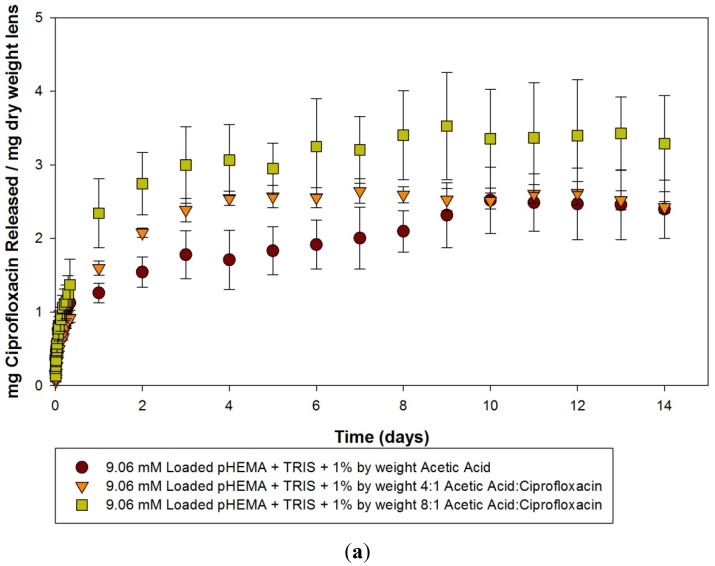

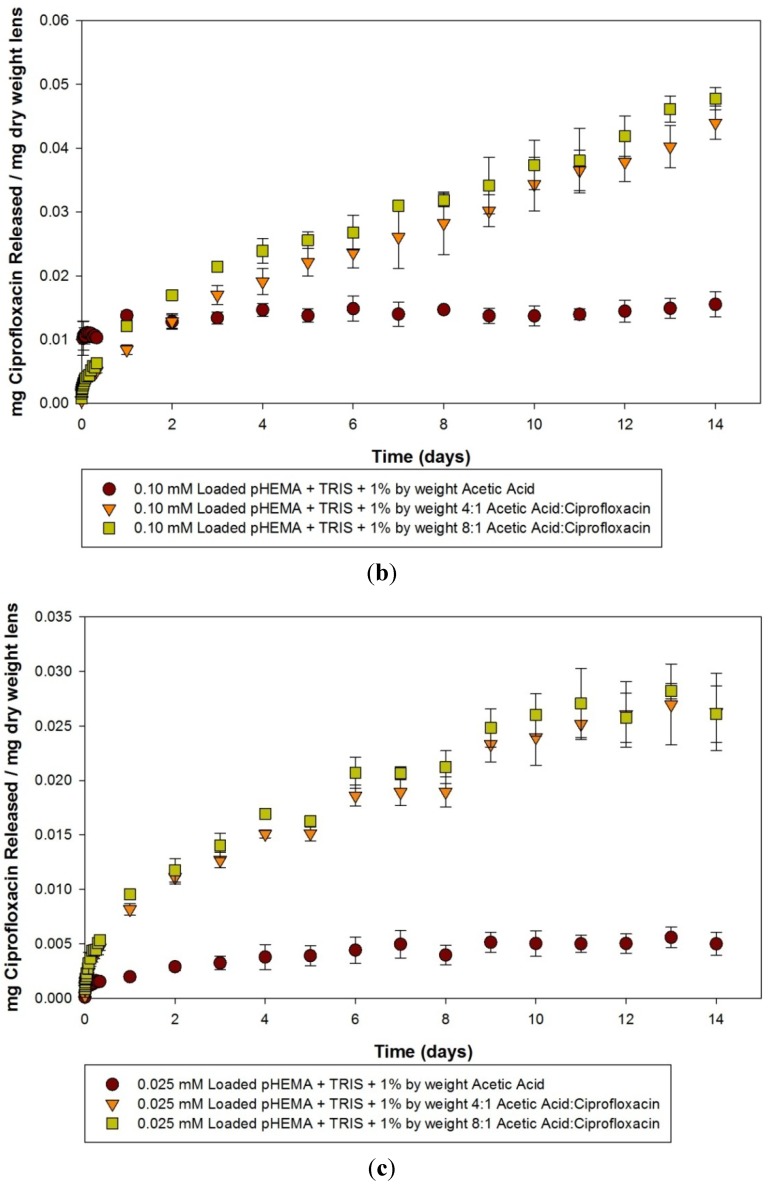
(**a**–**c**) Release curves from acetic acid imprinted materials loaded with (**a**) 9.06 mM ciprofloxacin; (**b**) 0.10 mM ciprofloxacin and (**c**) 0.025 mM ciprofloxacin over 14 days. Values plotted are means ± SD.

The release curves of the acetic acid imprinted materials and controls after 14 days of release is seen in Figure 2(a–c). For model lenses loaded with 9.06 mM of ciprofloxacin, there was an overall statistically significantly greater amount of drug released by the imprinted materials compared to the control (*p* < 0.05), but there was no significant difference between the two imprinted materials (*p* > 0.05). The time to reach the plateau was also different; interestingly, the imprinted materials appeared to reach their plateaus within 4 or 5 days, while the statistics suggest that the control was releasing for up to 8 days. Unfortunately, there is a greater amount of variation in the determination of the concentration of ciprofloxacin within the solution when loading with such a high concentration, as dilutions are necessary to reach concentrations relevant to the linear portion of the standard curve, potentially confounding results. The effect of imprinting in comparison with the non-imprinted controls is most evident again when the materials are loaded with the lower concentration solutions (0.10 mM and 0.025 mM), as seen in Figure 2b and Figure 2c. Here, the imprinting demonstrates two key advantages over the non-imprinted control, with a longer release time and a greater amount of ciprofloxacin being released. For the 0.10 mM loaded materials, analysis suggests that a plateau level is reached in as little as 45 min for controls. In contrast, the 4:1 imprinted and 8:1 imprinted materials demonstrate continued significant release compared to earlier time points out to 10 days. Similar results are seen in model lenses loaded with 0.025 mM solutions. The control released so little that there was statistically no difference over the course of the 14 days compared to the initial time point, whereas the imprinted materials were releasing for up to 8 days. As can be clearly seen from the release curves, there was no statistically significant difference between the two ratios of acetic acid to ciprofloxacin in terms of the plateau amount of ciprofloxacin released, or the time to reach a plateau.

The results from these initial attempts to create imprinted silicone hydrogel materials were very encouraging in that they achieved two separate goals. First, the effect of the imprinting was demonstrated when the model lenses were loaded with lower concentrations of the drug, as there was a clear difference between the imprinted and non-imprinted materials in their ability to deliver drugs for an extended period of time, as evidenced by drug release occurring for a period of 8 to 10 days (depending on the loading concentration). Second, we were able to confirm the delivery of relevant amounts of the antibiotic. When loaded with the clinical concentration of ciprofloxacin (9.06 mM), concentrations were achieved in the 2 mL reaction vial that were clinically relevant in achieving the minimum inhibitory concentration (MIC_90_) of common ocular isolates [31]. Not surprisingly, when the loading concentration was decreased by approximately 100 times, the amount of drug released was less, and the MIC_90_ only reached concentrations relevant to more susceptible bacteria. Finally, the pilot study failed to demonstrate any differences between the ratio of acetic acid to ciprofloxacin used to create the imprinting that has been demonstrated previously [2,16,17,19,21]. This was possibly due to the lack of precision in choosing to add the imprinting mixture on the basis of percentage weight rather than by molar concentration of the functional monomer, in relation to the number of moles of the other components of the polymerization as a whole.

### 2.2. Ciprofloxacin pHEMA-TRIS Materials with Acrylic Acid Functional Monomers

To further explore the effect of imprinting on the model silicone hydrogel materials, a second, larger study was conducted with a few key modifications to the imprinting process. The overall functional monomer concentration within the polymerization mix was varied between two concentrations (100 mM and 200 mM), and the functional monomer was changed to a related molecule, acrylic acid, which has had some success in the literature in terms of efficiently creating imprinted cavities [20]. The same three loading concentrations were used, and three separate imprinted ratios of acrylic acid to ciprofloxacin were used: 4:1, 8:1 and 16:1. The dry weight (g) and the water content (%) of the created materials are listed in Table 2. Similar to the model materials, the majority of the model materials were of low water content, and some degree of modification would be necessary to increase the water content if these materials were to be used on the human eye. A one way ANOVA revealed a significant difference between the dry weights and water contents of the materials (*p* < 0.05). Post Hoc Tukey tests revealed that this difference was mainly confined to two model—the pHEMA + TRIS + 200 mM Acrylic Acid, 8:1 ratio to ciprofloxacin and the pHEMA + TRIS + 200 mM Acrylic Acid, 4:1 ratio to ciprofloxacin were found to be statistically different than the other model lens materials (*p* < 0.05).

**Table 2:** Dry weight and water content of acrylic acid imprinted pHEMA+TRIS materials.

Model lens type	Dry weight (g) (Average ± SD)	Water content (%) (Average ± SD)	Centre thickness (mm) (Average ± SD)	Volume (mm^3^) (Average ± SD)
pHEMA + TRIS + 100 mM Acrylic Acid Control	0.0417 ± 0.0058	16.8 ± 4.1	0.96 ± 0.07	75.5 ± 6.0
pHEMA + TRIS + 200 mM Acrylic Acid Control	0.0454 ± 0.0064	15.1 ± 1.8	1.05 ± 0.16	82.2 ± 12.6
pHEMA + TRIS + 100 mM Acrylic Acid, 4:1 ratio to ciprofloxacin	0.035 ± 0.0076	16.2 ± 3.6	0.78 ± 0.16	60.87 ± 12.3
pHEMA + TRIS + 200 mM Acrylic Acid, 4:1 ratio to ciprofloxacin	0.0576 ± 0.011	12.6 ± 2.2	1.13 ± 0.3	88.3 ± 23.6
pHEMA + TRIS + 100 mM Acrylic Acid, 8:1 ratio to ciprofloxacin	0.0428 ± 0.0054	14.5 ± 2.1	1.01 ± 0.12	79.6 ± 9.3
pHEMA + TRIS + 200 mM Acrylic Acid, 8:1 ratio to ciprofloxacin	0.0397 ± 0.010	17.7 ± 3.6	0.97 ± 0.22	75.8 ± 17.4
pHEMA + TRIS + 100 mM Acrylic Acid, 16:1 ratio to ciprofloxacin	0.0497 ± 0.0053	14.3± 2.2	1.13 ± 0.13	88.6 ± 9.9
pHEMA + TRIS + 200 mM Acrylic Acid, 16:1 ratio to ciprofloxacin	0.0523 ± 0.0062	13.6 ± 1.4	1.20 ± 0.14	94.6 ± 10.9

**Figure 3:**
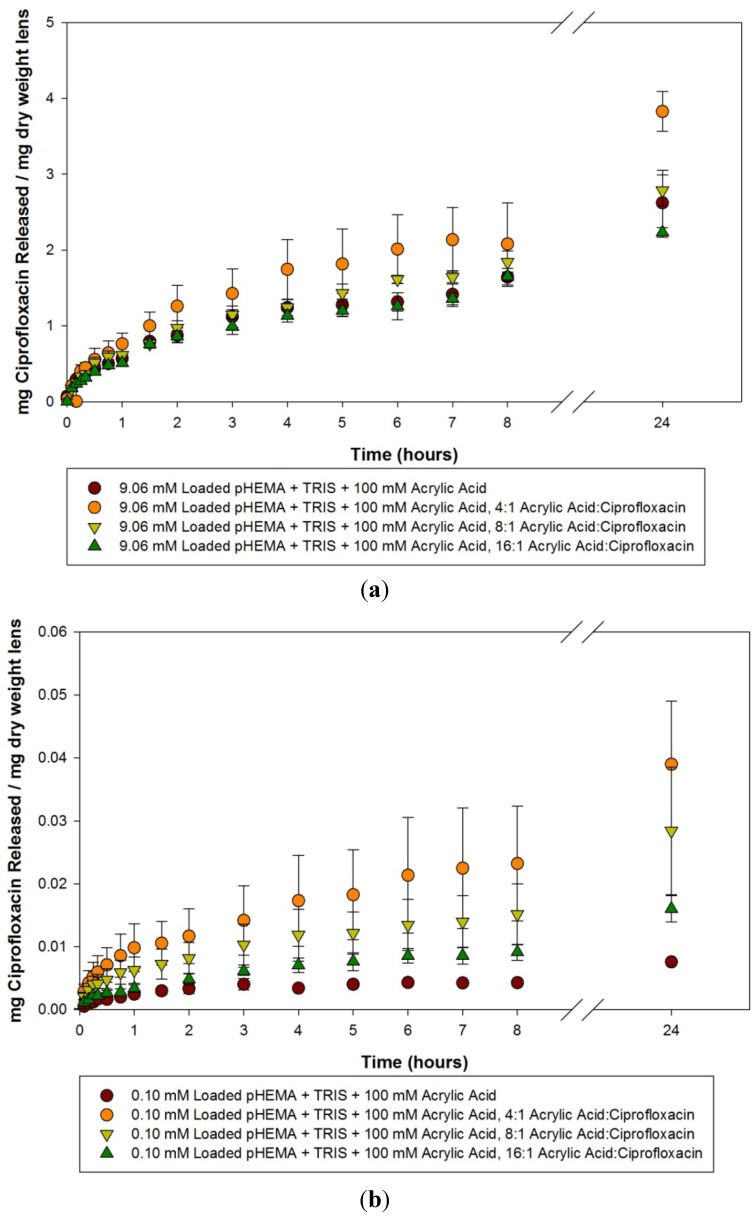

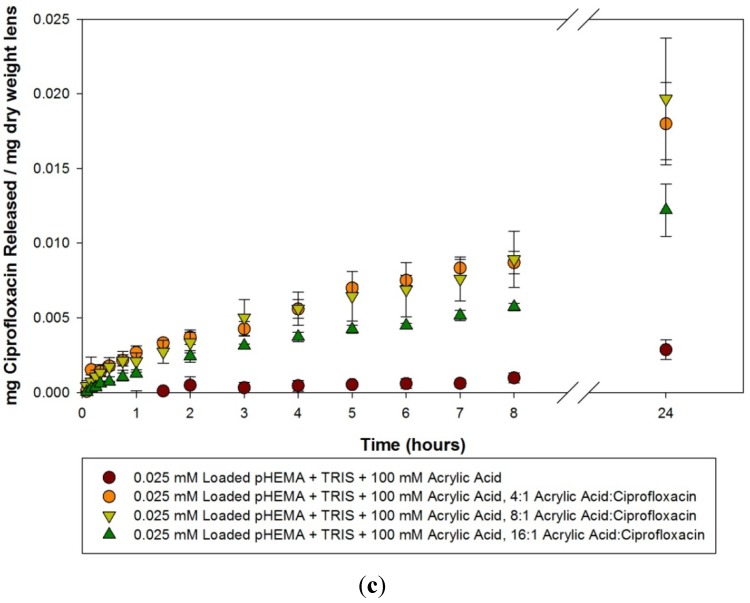
(**a**–**c**) Release curves from 100 mM acrylic acid imprinted materials loaded with (**a**) 9.06 mM ciprofloxacin; (**b**) 0.10 mM ciprofloxacin and (**c**) 0.025 mM ciprofloxacin over 24 hours. Values plotted are means ± SD.

**Figure 4:**
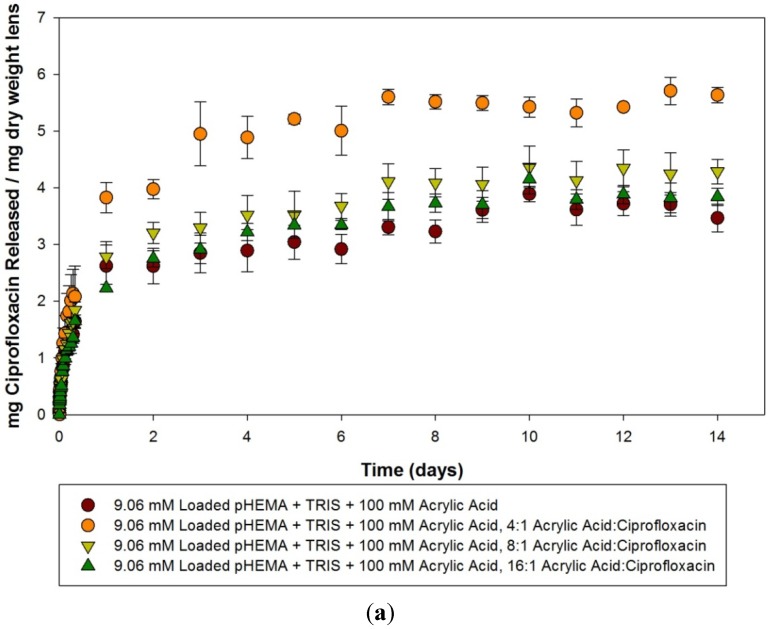

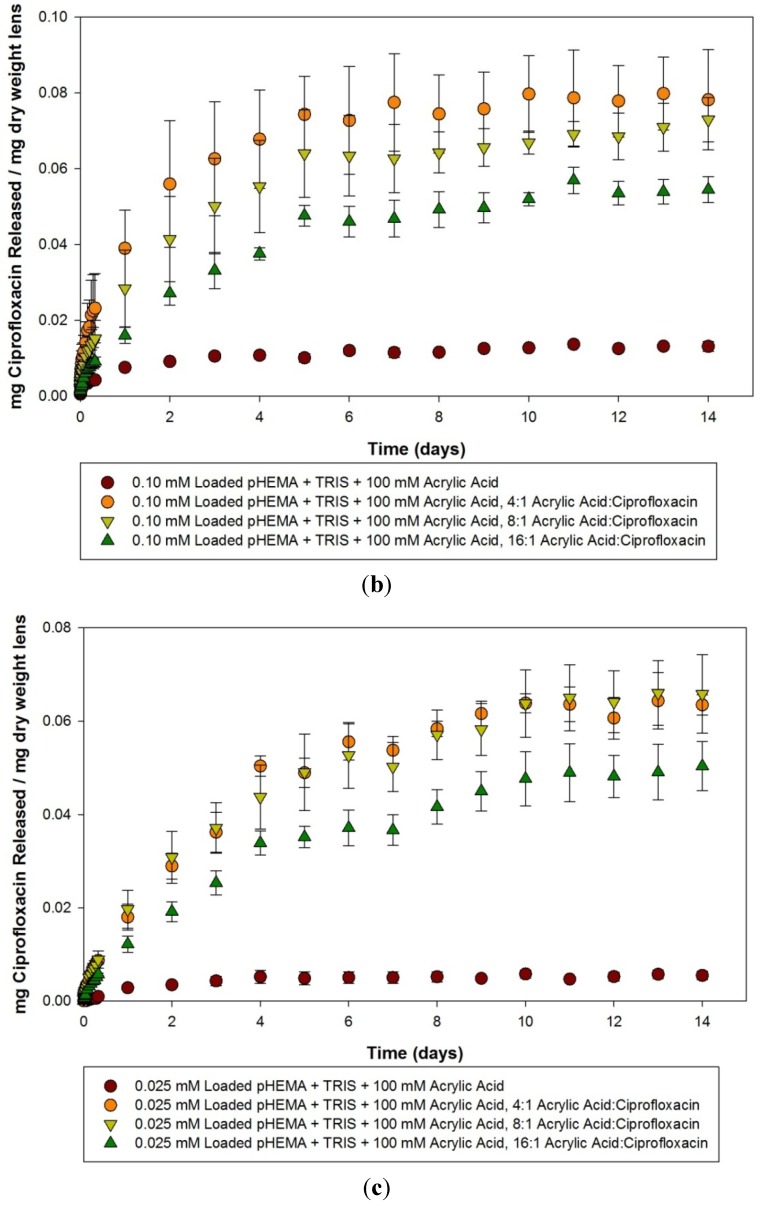
(**a**–**c**) Release curves from 100 mM acrylic acid imprinted materials loaded with (**a**) 9.06 mM ciprofloxacin; (**b**) 0.10 mM ciprofloxacin and (**c**) 0.025 mM ciprofloxacin over 14 days. Values plotted are means ± SD.

Ciprofloxacin release curves from 100 mM acrylic acid materials loaded with 9.06, 0.10 and 0.025 mM of ciprofloxacin within the first 24 h are detailed in Figure 3(a–c). A similar trend to that seen with the acetic acid imprinted materials is seen, as there are little differences in the amount or the rate at which ciprofloxacin was released from the 9.06 mM loaded model lenses, but when the materials were loaded with progressively lower amounts of ciprofloxacin the difference between the imprinted and the non-imprinted control became more apparent, with the imprinted materials releasing relatively more and at a greater rate. The release curves from these materials over the course of two weeks are detailed in Figure 4(a–c). Analysis of the model lenses loaded with 9.06 mM ciprofloxacin (Figure 4a) showed that the control model lens was only releasing for a maximum of 3 days before reaching a plateau, while the imprinted materials were releasing for periods up to 7 days. At plateau, the materials with 4:1 imprinting were found to be statistically significantly higher than the other model lens types (*p* < 0.05). The other model lens types (including the control) tended to cluster together. Analysis of the 0.10 mM loaded materials showed no significant release compared to the initial time point for the control, and significant release from the imprinted materials for up to 14 days in the case of the 8:1 imprinted material. The 16:1 imprinted material was found to be different from the other two imprinted materials (*p* < 0.05), while releasing for 11 days. The 4:1 imprinted materials released the most drug, but for the shortest period of time, at only 5 days. For materials loaded with 0.025 mM ciprofloxacin, the results were similar but with more extended release times. The 4:1 and 8:1 model lenses tended to cluster together and release the most amount of drug, while the 16:1 was statistically significantly lower, but still higher than the control (*p* < 0.05). All of the imprinted materials in this case took 10 days to reach a plateau level.

**Figure 5:**
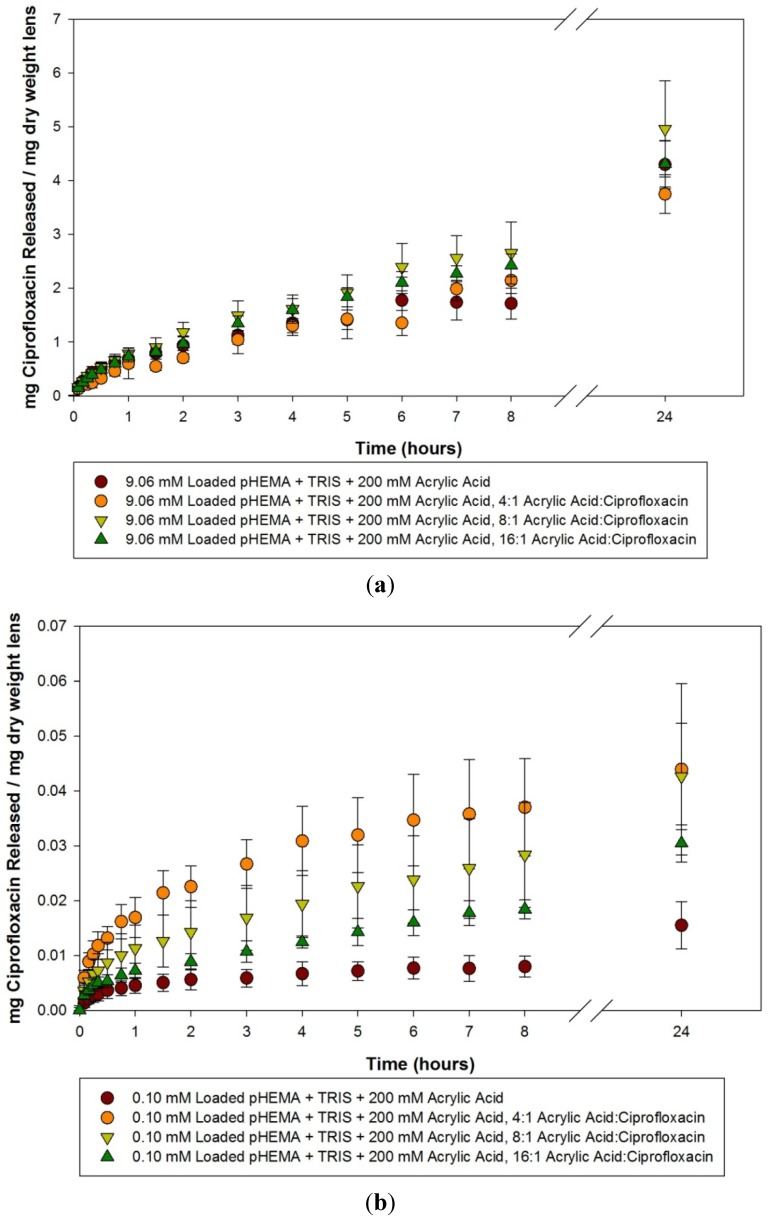

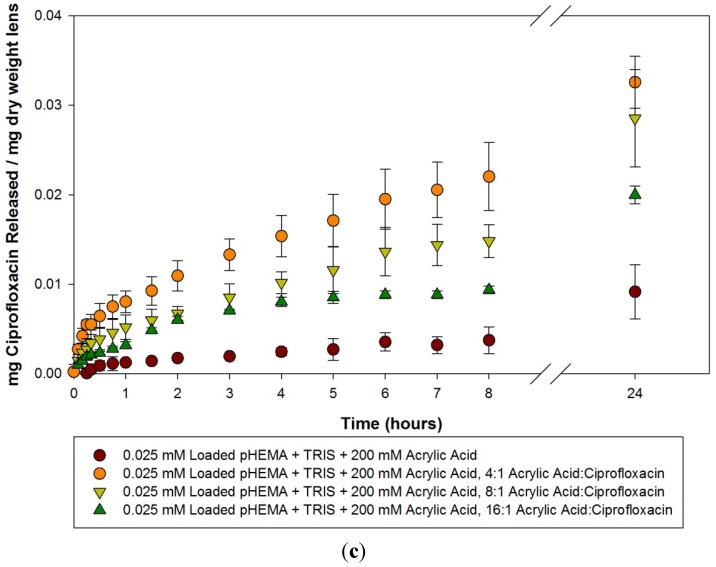
(**a**–**c**) Release curves from 200 mM acrylic acid imprinted materials loaded with (**a**) 9.06 mM ciprofloxacin; (**b**) 0.10 mM ciprofloxacin and (**c**) 0.025 mM ciprofloxacin over 24 hours. Values plotted are means ± SD.

Ciprofloxacin release curves from 200 mM acrylic acid imprinted materials loaded with 9.06 mM, 0.10 mM and 0.025 mM ciprofloxacin solutions for the first 24 h is presented in Figure 5(a–c). The loading of the high concentration (9.06 mM) led to all materials releasing a significant amount of drug, but there was no difference between the imprinted materials and the control (*p* > 0.05) over the first 24 h. For the model lenses loaded with 0.10 mM and 0.025 mM, the imprinted materials released a larger amount and at a faster rate compared to the control (*p* < 0.05), but there was no difference between the imprinted materials, although it appeared that the 4:1 loaded materials released more than the 8:1, and the 16:1 imprinted material released the lowest amount.

**Figure 6:**
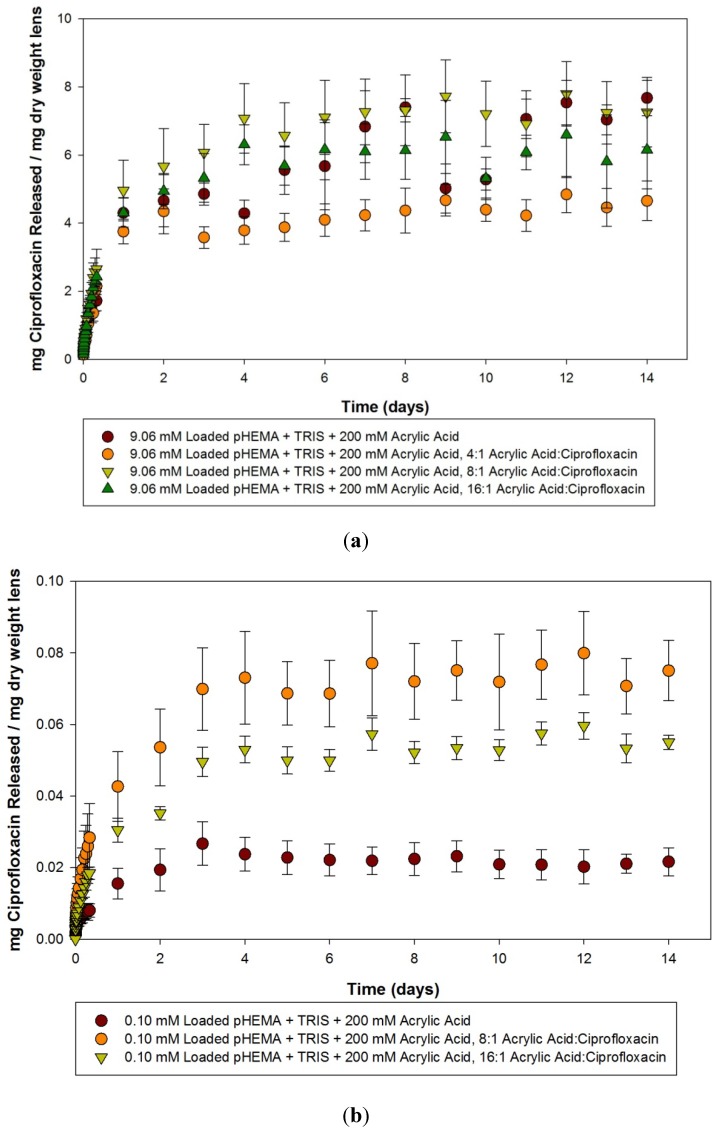

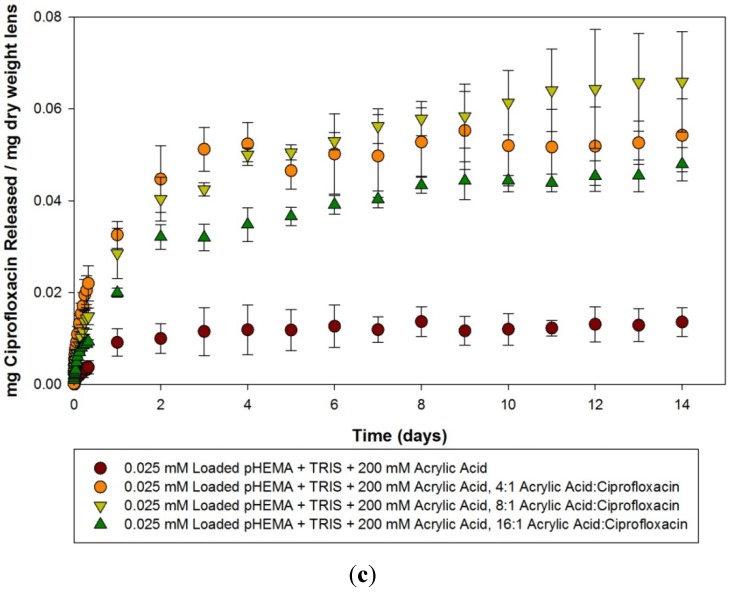
(**a**–**c**) Release curves from 200 mM acrylic acid imprinted materials loaded with (**a**) 9.06 mM ciprofloxacin; (**b**) 0.10 mM ciprofloxacin and (**c**) 0.025 mM ciprofloxacin over 14 Days. Values plotted are means ± SD.

Ciprofloxacin release curves from 200 mM acrylic acid imprinted materials loaded with 9.06 mM, 0.10 mM and 0.025 mM ciprofloxacin solutions over the course of 14 days is presented in Figure 6(a–c). The 9.06 mM loaded materials again showed a large amount of variation, and there was not statistically significant difference between the various imprinted materials versus the controls. The materials did release more than the required amount of antibiotic to be clinically relevant against common ocular pathogens. In the course of measurement over the two weeks, there was one anomalous group of readings. The 0.10 mm loaded, 4:1 imprinted materials began to show a declining concentration of ciprofloxacin within solution over time. Whether this was due to contamination, or drug degradation is unknown, regardless, the data is not presented here. Examination of the other 0.10 mM loaded materials shows that the imprinted materials released for up to 4 days, significantly different than the control (*p* < 0.05). The 0.025 mM loaded model lenses demonstrated significant differences between the 4:1 loaded and the other imprinted materials and the control, although the release time was relatively short at only 2 days. The 8:1 and 16:1 imprinted materials released comparatively less ciprofloxacin, but released it for longer periods of 13 and 14 days respectively. The control material loaded with 0.025 mM in comparison released relatively little ciprofloxacin over the course of 4 days, before no further changes were measured.

Thorough examination of the acrylic acid imprinted materials leads to several conclusions. The loading concentration of ciprofloxacin has a large role on the ability to detect the effect of the molecular imprinting. When the model lenses are loaded with a large concentration (9.06 mM), which is equivalent to the concentration of ciprofloxacin in commercially available 0.3% eye drops, there is little to no difference in the various imprinted materials and the controls. In this situation, it is likely that the majority of the ciprofloxacin was loaded into the material through non-specific concentration gradients, and the release from all the materials reflected that. One cannot discern the effect of the need for dilution to generate readings in the range of the linear standard curve as this could potentially affect the sensitivity to detect subtle changes in concentration within the solution, and may have contributed to the variability. However, this effect would be minimal.

When loaded with lower concentrations of ciprofloxacin, a different picture emerges from the data, in that the effect of imprinting these materials with template and the functional monomer become apparent. The imprinted materials release a larger amount compared to similarly loaded control materials, and for a significantly longer time. Release times for up to 14 days were seen in some cases, such as the 0.025 mM loaded, 200 mM acrylic acid 8:1 imprinted material, while control materials were confined to minimal release amounts for periods of only a few days. Interestingly, there was little to no difference between materials created with the two different concentrations of acrylic acid in terms of the amount or rate of ciprofloxacin being released. There has been some evidence in the literature that not only is the functional monomer:template ratio important, but so is the functional monomer:cross linker ratio [32]. In this experiment, there was no variation in the amount of crosslinker chosen, which was ethylene glycol dimethacrylate (EGDMA), so it would be interesting to see if the drug release rate dependence on functional monomer to crosslinker ratio would prove to be important in this model silicone hydrogel-type system.

In comparison with the pilot study, the functional monomer was changed to acrylic acid, and the precision to which the imprinting process was performed was more carefully controlled. In doing so, greater differences in the imprinted materials were demonstrated, with materials imprinted with the 4:1 ratio in general releasing the greatest amount of drug, with decreasing release from 8:1 and 16:1 imprinted materials respectively. This is similar to the results that were seen in a previous paper imprinting norfloxacin, another fluoroquinolone antibiotic [19].

The majority of the model lenses released enough antibiotic to reach concentrations that were clinically relevant for common bacterial isolates, especially with model lenses loaded with the clinical concentration of ciprofloxacin [31]. The difficulty is that sustained release over time was really only observed when loading with much lower concentrations, which can pose a problem with antibiotic therapy in preventing the development of bacterial resistance. To combat this, future studies should use newer and more potent antibiotics, whose minimum inhibitory concentrations are much lower than ciprofloxacin, such as the fourth generation fluoroquinolones moxifloxacin and gatifloxacin [33]. The challenge for these contact lens combination devices, especially antibiotic ones, beyond the demonstrated ability to sustain drug release, is acceptance into clinical practice. Considering the perception of the role of contact lenses in the etiology of severe ocular infections, use of a contact lens in such a situation faces an uphill climb in acceptance, and it will be the challenge to researchers and companies marketing such products to demonstrate advantages of such a device over traditional therapy.

The results from this study were generated using what is commonly known as the “infinite sink” technique, in which the release of drug is into the same static solution over time. This clearly does not necessarily mimic the ocular surface, in which tear production, evaporation and drainage can play a significant part in drug residence time and ultimately bioavailability to the cornea. The use of a static solution can also have a significant effect on release times for a drug such as ciprofloxacin, which is poorly soluble at physiological pH, potentially limiting release times due to the drug reaching a maximum soluble concentration within the solution. Several authors have proposed different solutions to this infinite sink problem. The simplest is to transfer the lenses to fresh solutions free of any drug at various time points, and sum up the release from all these release solutions [34]. A more sophisticated solution involves creation of an ocular tear flow device, in which the flow into, and drainage out of a tear solution as it interacts with the drug delivery device is controlled to mimic ocular tear flow. When such a system is used, authors have found that release rates are much slower than in infinite sink conditions, which is probably due to significantly smaller volumes of solution available to the device at any one given time. The release was also shown to follow zero order kinetics [22], and it would be interesting to test the materials created in this study under such conditions to observe any changes in release kinetics.

## 3. Experimental Section

### 3.1. Materials

2-Hydroxyethyl methacrylate (HEMA), ethylene glycol dimethacrylate (EGDMA), acrylic acid, acetic acid, and ciprofloxacin-HCl were purchased from Sigma-Aldrich (Oakville, ON, Canada). Methacryloxy propyl tris (trimethylsiloxy) silane (TRIS) was purchased from Gelest (Morrisville, PA, USA). IRGACURE was purchased from CIBA (Mississauga, ON, Canada). The HEMA and TRIS monomers were purified of the polymerizer inhibitor 4-methoxyphenol (MEHQ) by passing through an Aldrich inhibitor removers (Sigma-Aldrich). All other materials were not modified and used as obtained.

#### 3.2.1. Model Silicone Hydrogels

Model silicone hydrogel materials were created using a UV induced polymerization process. 3.6 g of HEMA was mixed with 0.4 g of TRIS. 0.2 g of EGDMA was subsequently added, allowed to mix, and finally 0.02 g of the photoinitiator IRGACURE was added. The mixture was poured into aluminum foil molds, and cured in a UV chamber (CureZone 2 Con-trol-cure) for 20 min at 340 nm. The surfaces were then placed in a 50 °C oven overnight to ensure completion of polymerization. Samples were then placed in Milli-Q water for a minimum of two days to rehydrate, with the water being changed daily to remove any unreacted monomers [35].

#### 3.2.2. Molecular Imprinted Materials—Acetic Acid Functional Monomer

Acetic Acid imprinted materials were created using a similar process to the model silicone hydrogels. To each polymerization mix before the addition of the IRGACURE initiator, acetic acid solution with various amounts of ciprofloxacin dissolved within it were added to the reaction mixture, creating an approximate 0.01 M acetic acid concentration in the final polymerization mixture. Control materials had a solution of acetic acid added without any ciprofloxacin.

#### 3.2.3. Molecular Imprinted Materials—Acrylic Acid Functional Monomer

The imprinting of acrylic acid materials was more carefully controlled to determine the effect of the imprinting on the drug release characteristics of the technique. To that end, materials were created using similar procedures to the model silicone hydrogels. Before the addition of the IRGACURE initiator, acrylic acid was added to a final concentration of either 100 mM or 200 mM. Ciprofloxacin powder was subsequently added to the mixture, in molar ratios to the acrylic acid varying from 1:4 to 1:16, and the polymerization of the materials was initiated as previous.

#### 3.2.4. Molecular Imprinted Materials—Washout

Materials imprinted with ciprofloxacin were rehydrated in Milli-Q water in glass jars, with the water being changed daily. The water used in the washout period was measured for ciprofloxacin concentration, and materials were only used after ciprofloxacin concentrations within the water were at minimal/non-existent levels.

### 3.3. Drug Solutions

A 0.3% (w/v) (9.06 mM) stock solution of ciprofloxacin-HCl was created in a phosphate buffered saline. The pH of the solution was adjusted to 4.0 to ensure the complete solubilization of the ciprofloxacin at this high concentration. Using this stock solution, samples were diluted approximately 4,000 times to be read by a Hitachi F-4500 fluorescence spectrophotometer (Hitachi Ltd., Tokyo, Japan), with an excitation wavelength of 274 nm and an emission peak at 419 nm to create a linear standard curve. This standard curve was used to correlate emission amounts with the concentration of ciprofloxacin within the solution.

### 3.4. Water Content, Centre Thickness, Volume and Dry Weight Determination

After soaking in Milli-Q water for a minimum of two days, discs of the materials were punched out using a #4 cork borer with a diameter of 5 mm. The water content of these discs was determined using the gravimetric method, using the Sartorius MV 100 (Sartorius Mechatronics Canada, Mississauga, ON, Canada). The dry weight of the disc was also determined. The centre thickness was determined using a dial lens gauge for rigid contact lenses (Vigor Optical, Carlstadt, NJ, USA), and the volume was calculated from thickness and diameter data, assuming a cylindrical shape.

### 3.5. Drug Loading into Materials

After determination of the water content, discs were placed in a ciprofloxacin drug loading solution. Three separate concentrations were used—the stock 9.06 mM, and two diluted loading concentrations, 0.10 mM and 0.025 mM. 2 mL of the loading solution was used, and this was undertaken in amber vials, as ciprofloxacin is light sensitive. Loading discs were left at room temperature for one week.

### 3.6. Drug Release Kinetics

Loaded discs were removed from the loading solution amber vials using plastic tweezers. The surface was partially dried on lens paper to remove any excess loading solution, and the disc placed into another amber vial containing 2 mL of an artificial tear solution (NaCl 90 mM, KCl 16 mM, Na_2_CO_3_ 12 mM, KHCO_3_ 3 mM, CaCl_2_ 0.5 mM, Na_3_Citrate 1.5 mM, Glucose 0.2 mM, Urea 1.2 mM, Na_2_HPO_4_ 24 mM, HCl 26 mM, pH 7.4) [36]. The vials were then placed in a shaking water bath at 34 °C. At various time points, the concentration of ciprofloxacin in the solution was determined using spectrophotometry. For model lenses loaded with 9.06 mM ciprofloxacin solution, samples were removed and diluted 100× to get into the range of the standard curve. For the other two loading conditions, 1 mL of the release solution was removed from the vial, read in the spectrophotometer, and returned to the vial. Readings were taken every 5 min for the first 20 min, then after 30, 45, 60 and 90 min. Readings were then taken hourly until 8 h had passed, then daily until 14 days had passed.

### 3.7. Statistical Analysis

Statistical analysis was performed using Statistica version 8 (StatSoft Inc., Tulsa, OK, USA) using a repeated measures ANOVA, and post hoc Tukey tests as indicated. A p value of less than 0.05 was considered statistically significant.

## 4. Conclusions

In this study, model silicone hydrogels for the delivery of the antibiotic ciprofloxacin were developed using a molecular imprinting strategy. Synthesized materials had water contents in the mid to low teens, and when loaded with various solutions of ciprofloxacin they demonstrated different release kinetics. Loading with high concentrations of ciprofloxacin led to very few differences in the various imprinted materials and the control. When loaded with lower concentrations, the effect of the imprinting was more clearly seen, with model lenses created using a 4:1 ratio of acrylic acid to ciprofloxacin template consistently releasing the greatest amount of drug, and certain model lenses continuing to release the drug for up to 14 days. As the use of these contact lens combination devices will likely involve some element of overnight or extended wear, the results from this study using model silicone hydrogel materials has provided some insight into how these materials behave as drug delivery devices when formed using molecular imprinting.
